# Group 2 Innate Lymphoid Cells (ILC2) Suppress Beneficial Type 1 Immune Responses During Pulmonary Cryptococcosis

**DOI:** 10.3389/fimmu.2020.00209

**Published:** 2020-02-14

**Authors:** Markus Kindermann, Lisa Knipfer, Stephanie Obermeyer, Uwe Müller, Gottfried Alber, Christian Bogdan, Ulrike Schleicher, Markus F. Neurath, Stefan Wirtz

**Affiliations:** ^1^Medizinische Klinik 1, Universitätsklinikum Erlangen, Friedrich-Alexander-Universität Erlangen-Nürnberg, Erlangen, Germany; ^2^Mikrobiologisches Institut - Klinische Mikrobiologie, Immunologie und Hygiene, Friedrich-Alexander-Universität Erlangen-Nürnberg, Erlangen, Germany; ^3^Centre for Biotechnology and Biomedicine, Institute of Immunology, College of Veterinary Medicine, University of Leipzig, Leipzig, Germany; ^4^Medical Immunology Campus Erlangen, FAU Erlangen-Nürnberg, Erlangen, Germany

**Keywords:** ILC2, fungi (Candida, *Cryptococcus*), innate immunity, lung infection, type 2 immune response

## Abstract

*Cryptococcus neoformans* is an opportunistic fungal pathogen preferentially causing disease in immunocompromised individuals such as organ-transplant-recipients, patients receiving immunosuppressive medications or, in particular, individuals suffering from HIV infection. Numerous studies clearly indicated that the control of *C. neoformans* infections is strongly dependent on a prototypic type 1 immune response and classical macrophage activation, whereas type 2-biased immunity and alternative activation of macrophages has been rather implicated in disease progression and detrimental outcomes. However, little is known about regulatory pathways modulating and balancing immune responses during early phases of pulmonary cryptococcosis. Here, we analyzed the role of group 2 innate lymphoid cells (ILC2s) for the control of *C. neoformans* infection. Using an intranasal infection model with a highly virulent *C. neoformans* strain, we found that ILC2 numbers were strongly increased in *C. neoformans*-infected lungs along with induction of a type 2 response. Mice lacking ILC2s due to conditional deficiency of the transcription factor RAR-related orphan receptor alpha (Rora) displayed a massive downregulation of features of type 2 immunity as reflected by reduced levels of the type 2 signature cytokines IL-4, IL-5, and IL-13 at 14 days post-infection. Moreover, ILC2 deficiency was accompanied with increased type 1 immunity and classical macrophage activation, while the pulmonary numbers of eosinophils and alternatively activated macrophages were reduced in these mice. Importantly, this shift in pulmonary macrophage polarization in ILC2-deficient mice correlated with improved fungal control and prolonged survival of infected mice. Conversely, adoptive transfer of ILC2s was associated with a type 2 bias associated with less efficient anti-fungal immunity in lungs of recipient mice. Collectively, our date indicate a non-redundant role of ILC2 in orchestrating myeloid anti-cryptococcal immune responses toward a disease exacerbating phenotype.

## Introduction

*Cryptococcus* (*C*.) *neoformans* is an opportunistic fungal pathogen that causes disease predominantly in immunocompromised individuals, such as organ-transplant-recipients, patients receiving immunosuppressive medications or, in particular, individuals suffering from HIV infection [reviewed in (1, 2)]. In these patients inhalation of the fungus, either in form of desiccated yeast cells or as spores, typically leads to a pneumonia-like illness. As a consequence of an exacerbating disease progression, the fungi have the propensity to pass the blood-brain-barrier causing life threatening cryptococcal meningitis [reviewed in (3)]. While *C. neoformans* exposure most likely occurs ubiquitously already during childhood, the vast majority of immunocompetent individuals completely clear the infection or control the pathogen asymptomatically in encapsulated cryptococcomas (4). Despite increasing incidence in immunocompetent patients (5, 6), the highest infection rates and disease manifestations are found in immunocompromised patients suffering from AIDS. Noteworthy, for the year 2014 more than 200,000 cases of cryptococcal meningitis, leading to more than 180,000 deaths (7).

Although a well-balanced regulation of the immune cell network protects from fatal outcomes in pulmonary cryptococcosis, the precise immunological mechanisms that direct the development of protective or detrimental anti-cryptococcal immunity are not clearly understood. However, numerous studies in mice clearly indicated that the control and clearance of *C. neoformans* is strongly reliant on prototypic type 1 immune responses, characterized by the production of inflammatory cytokines such as Interleukin (IL)-2, Tumor necrosis factor (TNF), and particularly Interferon (IFN)-γ as well as the activation of classical macrophages [reviewed in (8)]. Conversely, type 2 biased immune responses have been implicated in disease progression and detrimental outcomes (8). At mucosal surfaces, the release of alarmins such as IL-25, IL-33, and thymic stromal lymphopoietin (TSLP) was identified as important early trigger of type 2 immunity, which is defined by production of the cytokines IL-4, IL-5, and IL-13 [reviewed in (9–11)]. As a result, eosinophils and alternatively activated macrophages (AAMs) are activated and recruited, while goblet cell hyperplasia and T helper (T_H_)2 cell differentiation is induced (12). Indeed, detrimental *Cryptococcus*-induced type 2 immunity is characterized by elevated levels of the cytokines IL-4, IL-5, and IL-13, which seem to be important modulators of mucus hyperproduction, eosinophilia and AAM activation in the lungs of infected mice.

Recently, the immunomodulatory capacity of group 2 innate lymphoid cells (ILC2s) in the context of various infectious diseases became evident and ILC2s effector functions were considered as potentially important for shifting early immune responses toward a type 2 phenotype. ILC2s were shown to produce high amounts of type 2 cytokines upon activation by alarmin stimulation [reviewed in (10, 13, 14)]. It was recently demonstrated that the alarmin IL-33 is strongly induced during the development of pulmonary cryptococcosis. Because proliferation and activation of ILC2s are impaired in mice lacking the IL-33 receptor T1/ST2 (15, 16) and ILC2 effector cytokines such as IL-4, IL-5, and IL-13 exert critical effects in anti-cryptococcal immune responses (15–22), it is tempting to speculate that ILC2s may also play important roles during disease progression. However, direct effects of ILC2s during pulmonary *C. neoformans* infections still remain to be determined more precisely. Previous studies utilized IL-4R, T1/ST2, and IL17Rb/ST2 receptor knockout animals (16, 17) to address indirectly the involvement of ILC2s in the modulation of anti-cryptococcal immunity. However, in this broad approach, deficiencies of the immunological functions of AAMs, ST2^+^/Gata3^+^ T_H_2 cells, and ST2^+^ regulatory T cells most likely influenced the disease outcome, which essentially precluded firm conclusions on the role of ILC2s for the course of pulmonary *C. neoformans* infection.

In order to verify and substantiate the function of ILC2s during different phases of pulmonary cryptococcosis, we comprehensively studied the immune response in ILC2-deficient animals within the first 14 days of infection. Thereby, ILC2-deficient animals showed a massive downregulation of type 2 immunity, reflected by reduced levels of the type 2 signature cytokines IL-4 and IL-13. Importantly, ILC2-deficiency in mice was associated with an increase in type 1 immunity, increased pulmonary frequencies of classically activated macrophages, improved fungal control and prolonged survival. Collectively, these date corroborate a non-redundant role of ILC2 in orchestrating anti-cryptococcal immune responses toward a disease exacerbating phenotype.

## Results

### ILC2 Deficiency Is Associated With Decreased Fungal Burden and Reduced Pulmonary Damage

**Figure 1:**
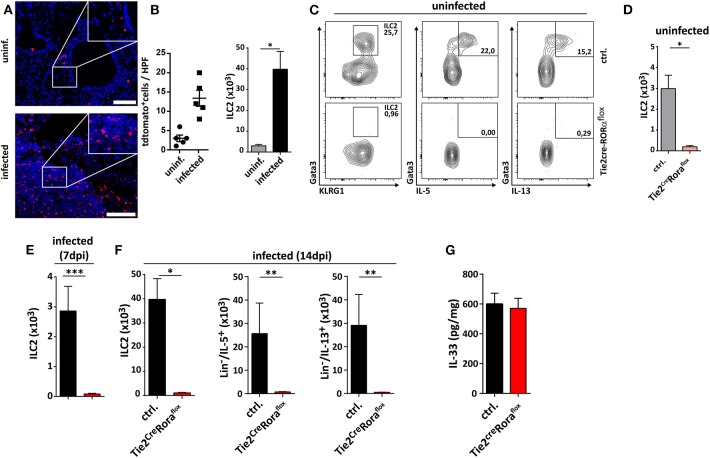
Analysis of ILC2s in lungs of *C. neoformans* infected mice. **(A,B,E–G)** Mice were infected intranasally with 500 cfu *C. neoformans*. and analyzed 7 dpi **(E)** or **(A,B,F,G)** 14 dpi. **(A)** Representative IHC-pictures of lungs of Rora^Cre^tdTomato^flox^ bone marrow chimeric mice and quantification of tdTomato expressing cells in naïve and *Cryptococcus*-challenged animals. **(B)** Pulmonary ILC2 counts of naïve and *C. neoformans* infected C57BL/6 mice (*n* = 4–5/group). **(C)** Representative flow cytometric plots of lung Lin^−^/Thy1^+^/Gata3^+^/KLRG1^+^ ILC2, Lin^−^/Thy1^+^/Gata3^+^/IL-5^+^ ILC2 and Lin^−^/Thy1^+^/Gata3^+^/IL-13^+^ ILC2 of healthy controls and Tie2^cre^Rora^flox^ mice. **(D)** Quantification of naïve lung Lin^−^/Thy1^+^/ICOS^+^/KLRG1^+^ ILC2 in control (*n* = 4) and Tie2^cre^Rora^flox^ mice (*n* = 4). **(E,F)** Quantification of Lin^−^/Thy1^+^/ICOS^+^/KLRG1^+^ ILC2 (*n* = 4), Lin^−^/IL-5^+^ (ctrl. *n* = 4, Tie2^cre^Rora^flox^ mice *n* = 6), and Lin^−^/IL-13^+^ (ctrl. *n* = 4, Tie2^cre^Rora^flox^ mice *n* = 6) populations in *C. neoformans-*challenged animals. **(G)** IL-33 concentration in lung tissue quantified per mg of total protein in control (*n* = 18) and Tie2^cre^Rora^flox^ mice (*n* = 26). Data is shown as mean ± SEM for at least two independent experiments. *P ≤ 0.05; **P ≤ 0.01 and ***P ≤ 0.001 using a Mann-Whitney *U*-test.

Recently, the infection-dependent release of the alarmin IL-33 has been identified as one of the main initial events for establishment of a type 2 polarized immune response in murine cryptococcosis (15, 16, 23). Given the importance of extracellular IL-33 for activation of these cells, we aimed to address the role of pulmonary ILC2s in a well-established murine model of acute pulmonary cryptococcosis using the virulent *C. neoformans* serotype A strain ATCC 90112 (24). Therefore, we intranasally infected Rora^Cre^tdTomato^flox^ mice, in which ILC2s are marked by strong expression of the fluorescent reporter protein tdTomato due to their strong expression of the transcription factor *rora* (25). While only few tdtomato^+^ cells were detectable at steady state, strongly increased numbers of labeled cells were present in lungs 2 weeks after infection as evidenced by confocal microscopy (Figure 1A). In line, flow cytometric analysis also demonstrated ILC2 accumulation in lungs of mice upon cryptococcal challenge (Figure 1B). Next, we analyzed Tie2^Cre^Rora^flox^ mice, which due to deficiency of the transcription factor *rora* in hematopoietic cells display an almost complete deletion of steady state pulmonary IL-5- and IL-13-producing ILC2s on day 14 pi (Figures 1C,D), but show normal development of T cell subsets including regulatory and T_H_17 cells *in vitro* (Supplemental Figures 1A,B) (26). In contrast to control mice, cryptococcal challenge of Tie2^cre^Rora^flox^ mice did not result in increased accumulation of pulmonary ILC2s producing IL-5 and IL-13 on day 7 and 14 pi (Figures 1E,F). Similar results were obtained in lethally irradiated C57BL/6 mice reconstituted with bone marrow from Tie2^cre^Rora^flox^ mice (Supplemental Figures 1C,D). Notably, this outcome was not related to a differential presence of IL-33 protein (Figure 1G), a key cytokine responsible for directing early immunomodulatory pathways during the onset of pulmonary cryptococcosis (15).

**Figure 2:**
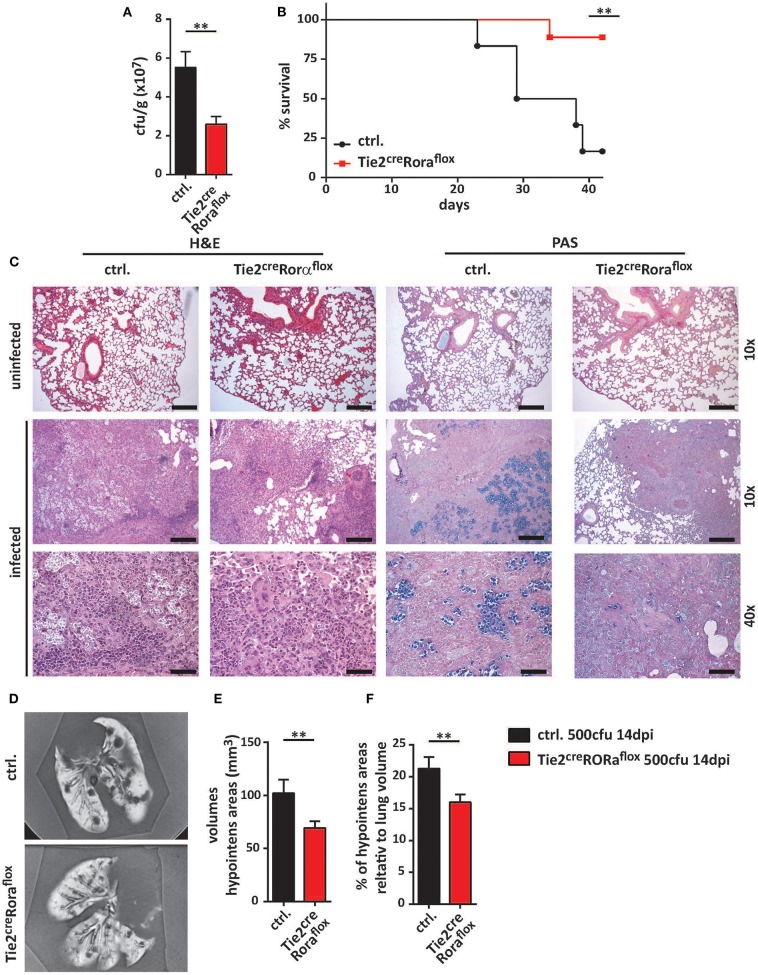
Improved fungal control in ILC2-deficient mice. **(A–F)** Control and Tie2^cre^Rora^flox^ mice were intranasally infected with 500 cfu of *C. neoformans* and sacrificed after 2 weeks. **(A)** Pulmonary fungal burden of control (*n* = 13) and Tie2^cre^Rora^flox^ mice (*n* = 17) was determined by plating serial dilutions of organ homogenates on SAB-Agar. **(B)** Animal fitness was judged over the indicated time course and illustrated as % survival. **(C)** Representative H&E and PAS stainings of paraffin-embedded lung sections. Scale bar 10× magnification = 200 μm, scale bar 40× magnification = 50 μm. **(D)** *Ex-vivo* MRI images of lungs were used to determine **(E)** total and **(F)** relative volumes of T2 weighted hypointense cryptococcal and inflammatory cell accumulations in infected control and Tie2^cre^Rora^flox^ mice (*n* = 10/group). Data is expressed as mean ± SEM, and pooled from **(B,D–F)** two or **(A)** three independent experiments **P ≤ 0.01 using a Mann-Whitney *U*-test.

In the next set of experiments, we analyzed whether ILC2-deficiency in Tie2^cre^Rora^flox^ mice affected their capacity to control *C. neoformans* infection. At 7 dpi, there were no major differences in pulmonary fungal burdens between Tie2^cre^Rora^flox^ mice and littermate controls (Supplemental Figures 2A,B). Strikingly, ILC2-deficient mice displayed significantly reduced cryptococcal burdens in lung tissues at 14 dpi compared to controls (Figure 2A). Moreover, the vast majority of the ILC2-deficient mice survived the infection for more than 40 days, while the infection-dependent lethality of control mice was substantially lower [mean survival of 33.5 days (Figure 2B)]. Furthermore, histologic assessment of hematoxylin/eosin (H&E) or Periodic acid-Schiff (PAS) stained lung tissue sections demonstrated that cryptococcal loads and the degree of tissue damage and mucosal inflammation was markedly reduced in the absence of ILC2s (Figure 2C). In line with this, magnetic resonance imaging (MRI) of whole lungs revealed a significantly higher presence of hypointense areas in infected control mice, which most likely represent local regions with concentrated fungal burden and strong immune cell infiltration (Figures 2D,E). Additionally, the volumes of hypointense areas relative to total lung volumes were significantly reduced in ILC2-deficient mice (Figure 2F). Further analyses also revealed that systemic spread and brain dissemination were tentatively lower in ILC2-deficient mice at 14 dpi as evidenced by analysis of liver and CNS fungal cultures (Supplemental Figures 2C,D).

**Figure 3:**
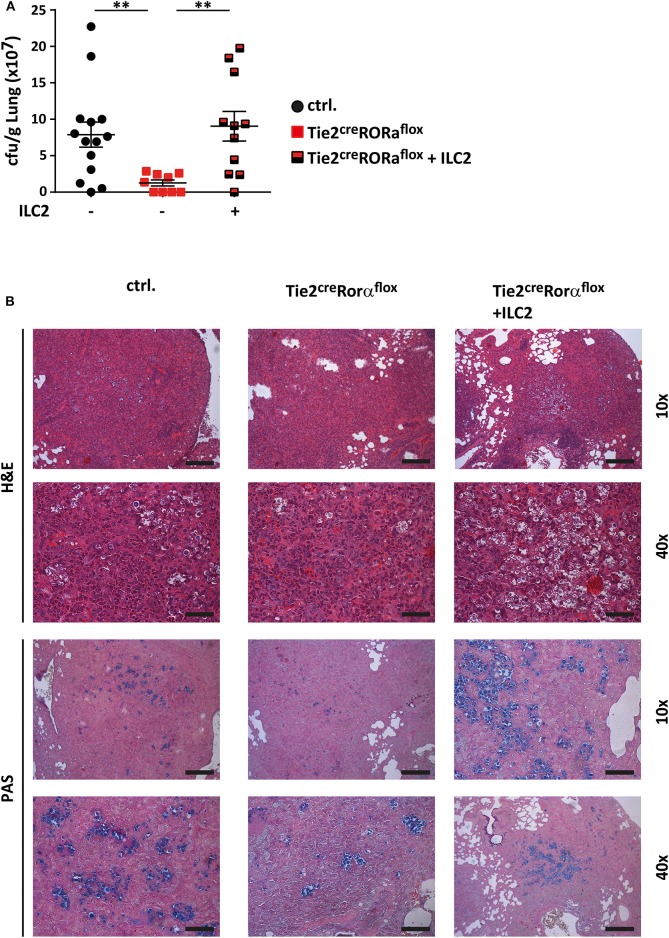
ILC2 drive detrimental anti-cryptococcal immune responses. **(A,B)** Control and Tie2^cre^Rora^flox^ mice were challenged with 500 cfu of *C.neoformans*. and analyzed 14 dpi. In addition, Tie2^cre^Rora^flox^ mice were reconstituted with sort-purified and *in vitro* expanded ILC2 on day 1 and 7 of infection as described in the method section (Tie2^cre^Rora^flox^ + ILC2). **(A)** Infection loads in affected lungs of ILC2 reconstituted Tie2^cre^Rora^flox^ mice (*n* = 11) were quantified and compared to control. (*n* = 14) and Tie2^cre^Rora^flox^ mice (*n* = 9). **(B)** Representative H&E and PAS stainings of control, Tie2^cre^Rora^flox^ and ILC2 reconstituted Tie2^cre^Rora^flox^ mice. Scale bar 10× magnification = 200 μm, scale bar 40× magnification = 50 μm. Data is expressed as mean ± SEM, and pooled from **(A)** three independent experiments **P ≤ 0.01 using a Mann-Whitney *U*-test.

To further ascertain the functional impact of ILC2 on the immune response to cryptococcal infection, we next adoptively transferred sort-purified and *in vitro* expanded ILC2s of C57BL/6 mice into *C. neoformans* infected Tie2^cre^Rora^flox^ mice on a C57BL/6 background (27). These ILC2s are able to target the murine lung upon intravenous injection as we have recently shown in a model of type 2 mediated lung inflammation (27, 28), albeit rather high numbers of cells are required for effective long term studies. Strikingly, reconstitution of the ILC2 compartment was found to be associated with significantly increased lung fungal burden at 14 dpi as revealed by cfu determination (Figure 3A) and histological staining compared to controls indicating that the immunomodulatory functions of ILC2s are sufficient to abrogate the protective phenotype in Tie2^cre^Rora^flox^ mice (Figure 3B).

Collectively, these data suggest a critical and detrimental role of ILC2s during the pathogenesis of murine pulmonary cryptococcosis.

### *C. neoformans*-Infected ILC2-Deficient Mice Display Reduced Pulmonary Type 2 Cytokine Production

**Figure 4:**
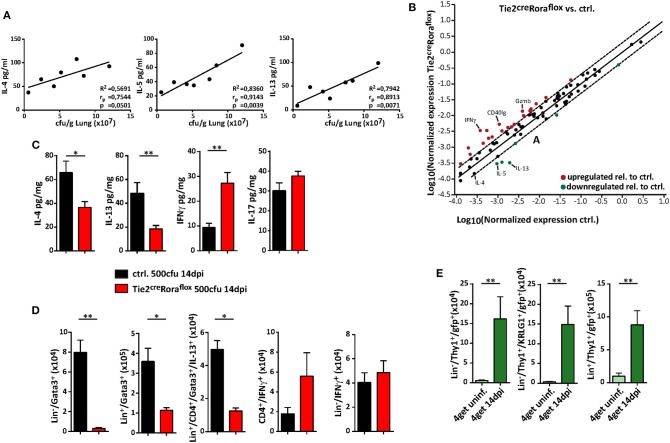
ILC2 orchestrate the local pulmonary cytokine milieu during cryptococcosis. **(A–D)** Control and **(B–D)** conditional Rora deficient mice (Tie2^cre^Rora^flox^) were subjected to intranasal *C. neoformans* infection (500 cfu infection dose) for 14 days. **(A)** Correlation analysis of cytokine concentrations (IL-4, IL-5, and IL-13) with fungal burden (*n* = 7). **(B)** Scatter plot of differentially regulated genes in Tie2^cre^Rora^flox^ compared to control mice. Data was generated by using RT^2^ Profiler PCR Array PAMM-074 (Qiagen) and relative gene expression was analyzed in the web-based Qiagen Data Analysis Center. Boundaries mark threshold of 2-fold change (*n* = 4, pooled/group). **(C)** Total lung protein concentrations of IL-4 (ctrl. *n* = 11; Tie2^cre^Rora^flox^ mice *n* = 18), IL-5 (ctrl. *n* = 9; Tie2^cre^Rora^flox^ mice *n* = 14), IFNγ (ctrl. *n* = 9; Tie2^cre^Rora^flox^ mice *n* = 14) and IL-17 (ctrl. *n* = 11; Tie2^cre^Rora^flox^ mice *n* = 18) was quantified in response to *C. neoformans* infection. **(D)** Lung single cell preparations were stimulated with PMA/ionomycin in the presence of protein transport inhibitors for 4 h. Cell numbers of Lin^−^/Gata3^+^ ILC2, Lin^+^/Gata3^+^ cells, Lin^+^/CD4^+^/Gata3^+^/IL-13^+^ T cells, CD4^+^/IFN-γ^+^ T cells, and Lin^−^/CD4^−^/IFN-γ^+^ ILC1 determined in lung tissue of infected mice (ctrl. *n* = 8; Tie2^cre^Rora^flox^ mice *n* = 11). **(E)** Analysis of naïve or *C. neoformans* infected (14 dpi) 4get mice. Data is presented as mean ± SEM **(A)** representative of two experiments, **(B)** one experiment or **(C–E)** pooled from 2 to 3 experiments. *P ≤ 0.05; **P ≤ 0.01 defined by Mann-Whitney *U*-test.

The quality of the cytokine response plays a significant role in protective anti-cryptococcal immunity, and early detection and control by the immune response is necessary to prevent systemic dissemination of this fungal pathogen. Indeed, increasing concentrations of the type 2 cytokines IL-4, IL-5, and IL-13 in lung homogenates positively correlated with cryptococcal burdens in our experimental settings (Figure 4A). We therefore next analyzed, to which extent ILC2-deficency in Tie2^cre^Rora^flox^ mice alters the pulmonary immune response upon cryptococcal infection. By profiling the expression of 84 T_H_ cell related genes by PCR array analysis, we found that total lung tissue homogenates of infected ILC2-deficient mice contained less transcripts of the type 2 cytokines IL-4, IL-5, and IL-13, while transcripts for the important T_H_1-related cytokine IFN-γ and the cytotoxic factor granzyme B were increased in these mice (Figure 4B). Accordingly, specific ELISA analysis of lung homogenates also demonstrated decreased concentrations of T_H_2 cytokines and increased presence of IFN-γ on protein level in lungs of infected Tie2^cre^Rora^flox^ mice. By contrast, the concentrations of the cytokine IL-17, which at least in some reports has strongly been implicated in pathways related to protective anti-fungal immunity (29, 30), were not significantly altered between the two experimental groups (Figure 4C). On a cellular level, both IL-13-producing ILC2 and to a lesser extent T_H_2 cells were reduced in Tie2^cre^Rora^flox^ mice at 14 dpi as determined by flow cytometry compared to controls. Conversely, infected lungs of Tie2^cre^Rora^flox^ mice contained more CD4^+^ T cells producing IFN-γ, while the numbers of IFN-γ^+^ ILCs were comparable between both groups (Figure 4D). Notably, the analysis of 4get reporter mice, which express enhanced green fluorescent protein (eGFP) from the 3′ UTR of the endogenous *Il4* gene, indicated that in addition to T cells, ILC2s could be readily identified as potential source of IL-4 in infected lungs (Figure 4E). Collectively, our data supports the importance of type 2 cytokine expression during the onset of pulmonary cryptococcosis and indicates that ILC2s critically contribute to a pathophysiologically relevant local cytokine milieu in the infected lung.

### ILC2-Deficient Mice Display Alterations in Myeloid Cell Composition and Macrophage Polarization During *C. neoformans* Infection

**Figure 5:**
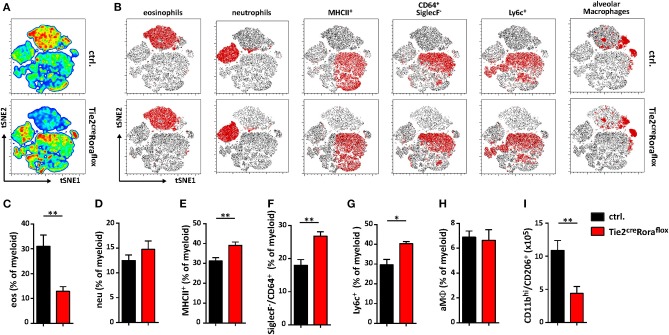
Altered polarization patterns in the myeloid compartment in the absence of ILC2 mice. **(A–I)** Control and Tie2^cre^Rora^flox^ mice were challenged intranasally with 500 cfu of *C. neoformans* and sacrificed 14 dpi. **(A)** t-SNE plots of flow cytometric analyzed pulmonary myeloid cell composition (t-SNE 1/t-SNE 2 represents individual marker expression of CD11b, CD11c, CD64, Ly6c, Ly6g, MHCII, and SiglecF of individual data points) in control and Tie2^cre^Rora^flox^ mice. Color code depicted is reflecting relative cell density (blue = low, red = high; *n* = 8/genotype). **(B)** Representative t-SNE-maps of control and Tie2^cre^Rora^flox^ mice show localization of respective surface marker positive cell clusters (red area) within t-SNE plots (*n* = 8). **(C–I)** Relative abundance of **(C)** CD11b^+^/CD64^−^/Ly6g^−^/SiglecF^+^ eosinophil Granulocytes (eos), **(D)** CD11b^+^/Ly6g^+^/Ly6c^+^ neutrophil Granulocytes (neu), **(E)** SiglecF^−^/MHCII^+^ cells, **(F)** SiglecF^−^/CD64^+^, **(G)** SiglecF^−^/Ly6c^+^ and **(H)** CD11b^int/+^/CD11c^int/+^/CD64^+^/SiglecF^+^ alveolar macrophages (aMϕ) referred to total myeloid cells in control (ctrl. *n* = 8) and Tie2^cre^Rora^flox^ (*n* = 7) mice. **(I)** Quantification of CD11b^hi^/CD206^+^ cells in total lung tissue of infected animals (ctrl. *n* = 4; Tie2^cre^Rora^flox^ mice *n* = 6). **(A,B)** t-SNE analysis and **(C–I)** flow cytometry shown is representative for two individual experiments. **(C,I)** Data is expressed as mean ± SEM. *P ≤ 0.05; **P ≤ 0.01 defined by Mann-Whitney *U*-test.

Among the multiple immune cell subtypes infiltrating *C. neoformans*-infected lungs, the myeloid cell lineage and in particular macrophages are of primary importance for pathogen control and eventual clearance. Because our data so far were indicative of strong shifts in the expression patterns of important cytokines previously reported to regulate the plasticity of these cells, we next aimed to comprehensively compare the myeloid compartment in infected ILC2-deficient mice and control mice by flow cytometry. Multivariate analysis using non-linear dimensionality reduction with stochastic neighbor-embedding approach (t-SNE) revealed striking differences in the pulmonary myeloid cell compartment in Tie2^cre^Rora^flox^ mice at 14 dpi (Figures 5A,B). Rather expectedly, numbers of eosinophils (CD11b^+^/CD64^−^/Ly6g^−^/SiglecF^+^), a cell type strongly dependent on IL-5 and eotaxin production by ILC2s (31), were massively reduced in ILC2-deficient mice at 14 dpi (Figure 5C). However, we did not observe a compensatory accumulation of CD11b^+^/Ly6g^+^/Ly6c^+^ neutrophils granulocytes (Figure 5D), which was described previously (32). Interestingly, mice lacking ILC2s displayed an increased abundance of SiglecF^−^/MHCII^+^ cells compared to controls (Figure 5E), suggesting changes in the monocyte/macrophage compartment. Detailed analysis of the monocyte/macrophage lineage revealed significantly increased frequencies of SiglecF^−^/CD64^+^ interstitial macrophages (Figure 5F) and SiglecF^−^/Ly6c^+^ monocyte-derived cells (Figure 5G) in the context of ILC2-deficency, while CD11c^int/+^/CD64^+^/SiglecF^+^ alveolar macrophages were seemingly unaffected (Figure 5H). Conversely, less myeloid cells expressing the mannose receptor (CD206) were present in lungs of mice lacking ILC2s indicating that the presence of ILC2s correlates to *Cryptococcus*-dependent accumulation of AAMs (Figure 5I). Notably, we observed no significant changes in myeloid cell populations at 7 dpi (Supplemental Figures 3A–F). We also compared the lung frequencies of dendritic cells (DCs). However, no significant changes were observed in the different DC subsets, including CD11c^+^CD103^+^ DCs, CD11c^+^CD11b^+^ DCs, and CD11c^+^Ly6C^+^ plasmacytoid DCs (Supplemental Figures 3G–I).

Collectively, these data support the notion that the presence of functional ILC2s is associated with compositional shifts in the myeloid cell compartment during pulmonary cryptococcosis.

## Discussion

Complex host-pathogen relationships characterize cryptococcal infections and an intricate balance between tolerance and resistance is required for effective pathogen control. There is a substantial body of literature that highlighted the critical role of T lymphocytes in the pulmonary immune response to *C. neoformans* challenge. Indeed, a number of studies have clearly emphasized the importance of T helper cell-mediated immunity in controlling cryptococcal infection. Thereby, resistance has been mainly associated to generation of polarized Th1/Th17 immune responses followed by STAT1-dependent activation of classical macrophages that ultimately facilitates fungal clearance (29, 33–35). Conversely, a dominance of type 2 immune responses and the activation of alternatively activated macrophages has been strongly linked to pathogen survival and systemic spread (36). However, with the recent description of ILCs as innate counterparts of T helper cells endowed with the potential to produce high amounts of important immunoregulatory cytokines, further research is warranted to address their relative contribution to anti-cryptococcal immunity. Here, we analyzed the role of ILC2s during pulmonary cryptococcosis using mice with specific deficiency in the ILC2 compartment. We observed that the numbers of ILC2 increased upon infection and that these cells were significant producers of type 2 signature cytokines. Notably, ILC2-deficiency was linked to increased pulmonary type 1 immunity and classical macrophage activation resulting in improved fungal control and prolonged survival, suggesting a detrimental role of ILC2 effector functions during *C. neoformans* infection.

Previous studies in Balb/c mice showed that the alarmin IL-33 derived from alveolar type 2 epithelial cells is an important trigger of lung type 2 cytokine production both at early and late phases of infection with a highly virulent *C. neoformans* strain (15, 16, 23, 37). While deficiency in IL-33 signaling attenuated infection-induced ILC2 numbers and their production of IL-13 and IL-5 in these studies, it remained unclear, whether ILC2s and their cytokine production significantly contributed to AAM differentiation and the severity of pulmonary disease. Using mice lacking ILC2s due to conditional deletion of the transcription factor Rorα in immune cells, we provide direct evidence that ILC2s functionally contribute to mechanisms of immune polarization during cryptococcosis. Although Rorα is not exclusively expressed in ILC2s, our data indicated that T helper cell subset differentiation was not affected in Tie2^Cre^Rora^flox/sg^ mice. Similarly, a broadly used strategy to delete Rorα using IL-7R^Cre^ deleter mice in CD127-expressing cells (e.g., T cells, ILCs, B cells) resulted in effective ILC2 depletion without obvious effects on Th2 cells during infection-dependent type 2-mediated lung inflammation (38).

Rorα deficiency in mice was reported to result in diminished Th17 responses *in vitro* and *in vivo* (39). However, we did not observe effects on the *in vitro* differentiation potential of naïve T cells toward Rorγt^+^ and IL-17 producing cells *in vitro*. Moreover, improved pathogen clearance in Tie2^Cre^Rora^flox/sg^ mice was not associated with changes in the frequencies of Th17 cells or IL-17 itself, which has been shown to protect from *C. neoformans*-induced lung pathology in some studies (29, 40). However, the role of IL-17 in cryptococcosis is controversial as in other studies IL-17 played no or only minor protective roles compared to IFN-γ-dependent classical macrophage activation (41, 42). In line with this, we observed no changes in the frequencies of lung infiltrating neutrophils in the absence of ILC2s in infected mice. Conversely, ILC2s were, most likely via the production of IL-5, essential for the pulmonary accumulation of eosinophils in our model. Notably, this ILC2-dependent increase in eosinophil numbers occurred in the presence of adaptive immune cells, while it was previously reported in Rag2^−/−^ mice that ILCs and NKs can drive eosinophilia even in the absence of T cells (32). Although eosinophilia has been associated with cryptococcal disease in mice and potentially humans (43–45), it remains unclear whether reduced eosinophil numbers in Tie2^Cre^Rora^flox/sg^ mice are directly linked to improved pathogen control and survival. Enhanced Th1 and Th17 responses were reported in *C. neoformans*-infected eosinophil-deficient ΔdblGATA mice, although lung fungal burdens and brain dissemination were similar to wildtype control mice (46). In the same study, Piehler et al. identified eosinophils as important producers of the cytokine IL-4, which is a key immunoregulatory factor in cryptococcosis (47). However, IL-4 production by eosinophils and antigen-specific Th2 cells occurred rather late during infection, suggesting that other IL-4 sources may contribute to Th2 polarization at early time points of the infection process. Using IL-4 reporter mice, we identified in the present study ILC2s as producers of IL-4 in infected lung tissue. Moreover, IL-4 transcripts were reduced in the context of ILC2-deficiency. Thus, IL-4 production by ILC2s may be positioned at the apex of a cascade leading to the development of Th2 polarization, while protective Th1 responses and the activation of classical macrophages are inhibited. Indeed, ILC2s have been shown to be substantial producers of IL-4 *in vivo* (26, 48). In addition, activated ILC2 are also well-known for their capacity to produce high amounts of IL-13, a cytokine with profound effects on macrophage cell biology in pathological microenvironments (49, 50). However, whether ILC2s directly via the production of prototypical cytokines or indirectly via other mechanisms such as direct cellular interaction contribute to macrophage polarization during *C. neoformans* still remains an open question. Further studies will have to dissect the individual contribution of ILC2 effector cytokines to T helper and macrophage polarization patterns in early *C. neoformans* infection.

In summary, we identified ILC2s as a critical regulatory cell lineage during pulmonary *C. neoformans* infections. Targeting ILC2s may therefore represent, in combination with other therapeutic strategies, a method to treat this severe inflammatory disease.

## Methods

### Animals and Husbandry

Tie2^cre^Rora^flox/sg^ (C57BL/6 background) were described previously (26, 27). Rora^Cre^tdTomato^flox^ mice were generated by crossbreeding Rora^Cre^ mice [kindly provided by Dennis O'Leary (51)] to ROSA26-LSL-tdTomato mice that contain a transgene encoding an enhanced tandem dimer tomato red fluorescent protein (tdTomato) in the ROSA26 locus with a lox-transcriptional stop-lox cassette inserted between exon 1 and exon 2 (52). 4get mice were kindly provided by David Vöhringer, Erlangen (53). C57BL/6 mice were purchased from The Jackson Laboratory. Animal husbandry and experiments were realized in accordance to the guidelines for “Tierhaltung Regierung Unterfranken” (accreditation no. 55.2-2532-2-827). Sterile drinking water and food were provided *ad libitum*. All animals were kept in individually ventilated cages (IVC), and the health status of the colony was assessed periodically for pathogens in adherence with the guidelines of the Federation of European Laboratory Animal Science Associations.

### Culture of *C. neoformans* and Infection Model

*C. neoformans* var. *grubii* (ATCC 90112) was thawed from glycerol stocks and cultured in Sabouraud Dextrose Broth (SAB-Medium; 2% Dextrose, 1% Peptone; Sigma-Aldrich) at 30°C overnight. Fungi were harvested and washed twice in sterile phosphate-buffered saline (PBS) and cell numbers were determined with the help of a Neubauer-improved counting chamber. Afterwards, suspensions containing 2.5 × 10^4^ cryptococci per ml in sterile PBS were prepared. Recipient mice were anesthetized intraperitoneally with 80 μl/10 g body weight of a ketamine/xylazine mixture [2.4 ml ketamine (50 mg/ml), 0.8 ml xylazine (20 mg/ml) in 6.8 ml sterile PBS] prior to intranasal application of a 20 μl inoculum to induce a low dose infection with 500 colony-forming-units (cfu). *C. neoformans* concentrations were verified by plating serial dilutions of the inoculum on Sabouraud Dextrose Agar (SAB-Agar; 4% dextrose, 1.5% agar, 0.5% casein, 0.5% animal tissue digest; Thermo Fisher Scientific) followed by incubation for 48 h at 30°C to determine the number of cfu. Infected mice were monitored daily with regard to their health status.

### Determination of Lung Fungal Burden

Lung, liver or brain tissue was removed, weighted and covered with 1 ml SAB-Medium per mg of tissue. Lung tissue integrity was disrupted by processing with a homogenizer (MM400 mixer mill, Retsch, Germany) with metal beads at a frequency of 25 Hz for 2 min. Serial dilutions were plated on SAB-Agar plates and incubated for 48 h at 30°C. The numbers of cfu/g tissue were determined under consideration of the dilution factor.

### Preparation of Pulmonary Leukocytes

For the preparation of lung leukocyte single cell suspensions, lung tissue was removed, rinsed in Hank's Balanced Salt Solution (HBSS; Sigma-Aldrich) and cut into small pieces. Subsequently, the lung tissue was digested in HBSS containing 0.2 mg/ml Liberase^TM^ (Roche) and 1 μg/ml DNase1 (2,000 U/mg; Roche) in a final volume of 2.5 ml. Digestion was performed with the help of a gentleMACS™ Octo Dissociator with heaters running the program 37C_m_LDK_1 (Miltenyi Biotec). Subsequently, the suspension was passed through a 70 μm nylon-mesh (Corning) and washed in phosphate buffered saline supplemented with 0.5% bovine serum albumin and 2 mM EDTA (PEB-Buffer). In order to remove erythrocytes, the suspension was spun down, resuspended in 1 ml ACK-Lysis-Buffer (155 mM ammonium chloride, 10 mM potassium bicarbonate, 0.1 mM EDTA) and incubated for 30 s. Subsequently, cells were washed in PEB-Buffer and passed to Percoll-centrifugation. In brief, 80% Percoll (GE Healthcare) was overlayed with 40% Percoll containing lung leukocytes. After centrifugation at 1,400 rpm for 20 min at room temperature without breaks, interphases were collected and washed in PEB-Buffer. Finally, cell numbers were determined in a Neubauer-improved counting chamber and single cell suspensions were used for further *ex vivo* phenotyping.

### Flow Cytometry

Lung leukocytes were incubated with anti-CD16/CD32 antibodies (anti-Fc-receptor; eBioscience) prior to specific surface marker and intracellular staining. For ILC2 identification, specific lineage preclusion was applied. In brief, Fc-receptor blocked cells were incubated with a custom made biotinylated lineage antibody cocktail (anti-B220, anti-CD3, anti-CD5, anti-CD11b, anti-GR1, anti-NK1.1, anti-SiglecF, anti-Ter119; Miltenyi Biotec). After washing, cells were passed to regular surface staining including streptavidin-Brilliant-Violet 421 (BV421; Biolegend) for labeling of biotinylated antibodies. Antibodies were purchased from Miltenyi Biotec if not otherwise indicated. APC (Alexa647), BV510, BV650, FITC, PacificBlue (BV421, eFluor410, VioBlue), PE, PE-Cy7 or PerCP-Cy5.5 (PerCP-efluor710, PerCP-Vio700) conjugated antibodies were used. For surface staining, cells were incubated with different combinations of anti-CD4, anti-CD64 (BD Bioscience), anti-CD11b, anti-CD11c, anti-CD103 (Biolegend), anti-CD206 (Biolegend), anti-ICOS anti-KLRG1, anti-Ly6c (BD Bioscience), anti-Ly6g (eBioscience), anti-MHCII (eBioscience), anti-SiglecF, and anti-Thy1.2 antibodies.

In order to enable intracellular cytokine staining cells were stimulated with the 1× Cell Stimulation Cocktail (plus protein transport inhibitors) (eBioscience) for 4 h. For subsequent intracellular staining of transcription factors and/or Interleukins, cells were fixed and permeabilized with the FoxP3 Transcription Factor Staining Buffer according to manufacturer's instructions (eBioscience). For intracellular staining, antibody combinations of anti-IL-13-PE (eBioscience), anti-IFNγ-APC (eBioscience), anti-Gata3-APC, anti-T-bet-PE (eBioscience), anti-RORγt-PE (eBioscience), and/or anti-FoxP3-PE (eBioscience) were utilized. Samples were analyzed on a LRSFortessa cell analyzer (BD Bioscience) and evaluated with Flowjo 10 (Treestar). For gating strategies of specific immune cell populations see Supplemental Figure 4.

t-SNE analysis was performed with Flowjo 10 (Treestar). In brief, FCS files of individual specimens of control and Tie2^cre^RORα^flox^ mice were down sampled randomly to 50,000 events per specimen and concatenated according to their originating genotype. t-SNE maps were generated by running the t-SNE analysis with following markers: CD11b, CD11c, CD64, Ly6c, Ly6g, MHCII, and SiglecF. Two dimensional reduction (t-SNE 1/t-SNE 2) was calculated by using the following parameters: 1,000 iterations, perplexity 30 and a learning rate (eta) of 56,000. Specific cell populations were defined according to their surface marker abundance.

### ILC2 Isolation and Expansion

For ILC2 induction *in vivo*, an IL-25 expression vector was hydrodynamically administered and murine spleen and mesenteric lymph nodes were isolated 3 days later (54). To generate single cell suspensions, tissues were digested in RPMI (Sigma-Aldrich), supplemented with 10% FBS (Gibco), 1% penicillin-streptomycin (Sigma-Aldrich), 0.25 mg/ml collagenase B (Roche) and 0.05 mg/ml DNase1 (2,000 U/mg; Roche) using the gentleMACS™ Octo Dissociator with heaters running the program 37C_m_SDK_1 (Miltenyi Biotec) according to manufacturer's instructions. After washing and cell number determination, cell suspensions were passed to fluorescence-activated cell sorting and ILC2 were identified using the following panel: CD3 FITC^−^ (17A2, BioLegend), CD5 FITC^−^ (REA421, Miltenyi Biotec), CD11b APC-Vio770^−^ (REA592, Miltenyi Biotec), CD11c APC-Vio770^−^ (N418, Miltenyi Biotec), CD45R FITC^−^ (REA755, Miltenyi Biotec), CD49b PE-Vio770^−^ (REA981, Miltenyi Biotec), FcεR1a PE-Cy7^−^ (MAR-1, Invitrogen), NK1.1 PE-Vio770^−^ (PK136, Miltenyi Biotec), ICOS VioBlue^+^ (7E.17G9, Miltenyi Biotec) and KLRG1 PE^+^ (REA1016, Miltenyi Biotec). FACS was performed using a MoFlo Astrios EQ (Beckman Coulter) in the Core Unit Cell Sorting Erlangen.

For *ex vivo* expansion of sort-purified ILC2, cells were cultivated in DMEM GlutaMax medium (Sigma-Aldrich) supplemented with 10% FBS (Gibco), 1% penicillin-streptomycin (Sigma-Aldrich), 20 mM Hepes (Carl Roth), 1 mM sodium pyruvate (Gibco), 50 μM mercaptoethanol (Sigma-Aldrich) together with 20 ng/ml TSLP (Invitrogen) and 50 ng/ml of IL-2 (BioLegend), IL-7 (Immunotools), IL-25 (Immunotools), IL-33 (Immunotools) each.

### Adoptive Transfer of ILC2 in *Cryptococcus neoformans*-Challenged Mice

ILC2-deficient Tie2^cre^Rora^flox^ mice were adoptively transferred with 1 × 10^7^ viable *in vitro* expanded ILC2 on day 1 and 7 of *C. neoformans* infection by intravenous administration. Thereafter, mice were sacrificed 14 dpi.

### Bone Marrow Chimeras

In order to generate bone marrow chimeric mice, lethally irradiated (10 Gray) C57BL/6 mice were reconstituted with 1 × 10^7^ femoral bone marrow cells of either control, Tie2^cre^Rora^flox^ or Rora^Cre^tdtomato^flox^ mice. Irradiated mice were kept in isolator cages and treated for 2 weeks with antibiotics (150 μl 24% Borgal® solution [200 mg sulfadoxine and 40 mg trimethoprim ad 1 ml aqua ad injectabilia; Virbac] in 300 ml drinking water). After 8 weeks of hematopoietic reconstitution, mice were challenged with *Cryptococcus neoformans*.

### T Cell Differentiation

Before *in vitro* T cell differentiation, splenic single cell suspensions were generated by passing spleens through a 40 μm nylon mesh (Corning), followed by a washing step in R10+ medium (see Table 1). Erythrocytes were removed in 5 ml ACK-Lysis-Buffer (155 mM ammonium chloride, 10 mM potassium bicarbonate, 0.1 mM EDTA) for 5 min. Thereafter, the cell suspension was washed in R10+ medium and cell number was determined. Isolation of naïve T cells was performed with mouse CD4^+^ T Cell Isolation and CD25 Isolation Kits (Miltenyi Biotec) according to manufacturer's instructions. For T cell differentiation, 1 × 10^6^ CD4^+^CD25^−^ T cells were seeded in 48-well plates coated with anti-CD3/anti-CD28 antibodies (BioXcell, 10 μg/ml each). The differentiation of the respective T cell population was induced by stimulating the cells according to Table 1 for 5 days.

**Table 1:** Stimulation media for T cell differentiation.

**Subpopulation**	**Reagent**	**Concentration**	**Medium**
T_H_0	/	/	R10+ (RPMI 1640 [with L-Glutamine and sodium bicarbonate, Sigma-Aldrich], 1% penicillin/streptomycin [Sigma-Aldrich], 10% FCS [Gibco])
T_H_1	anti-IL-4 rIL-12	5 μg/ml 20 ng/ml	R10+
T_H_2	anti-IFNγ anti-IL-12 rIL-2 rIL-4	5 μg/ml 10 μg/ml 20 ng/ml 100 ng/ml	R10+
T_H_17 (IL-23)	anti-IL-4 anti-IFNγ rIL-6 rIL-23 rIL-1β	5 μg/ml 5 μg/ml 100 ng/ml 50 ng/ml 20 ng/ml	X-Vivo+ (X-Vivo [Lonza] 1% penicillin/ streptomycin [Sigma-Aldrich])
T_H_17 (TGF-β)	anti-IL-4 anti-IFNγ rIL-6 rTGF-β	5 μg/ml 5 ng/ml 100 ng/ml 5 ng/ml	I10+ (IMDM [PAA]), 1% penicillin/ streptomycin [Sigma-Aldrich], 10% FCS [Gibco]
T_reg_	rTGF-β rIL-2	5 ng/ml 20 ng/ml	R10+

### Real-Time PCR

Lung tissue was snap-frozen in liquid nitrogen and stored at −80°C until RNA isolation. RNA was isolated using the RNeasy mini kit (Qiagen, Germany) according to manufacturer's instructions. cDNA was synthesized with the Script RT-PCR kit (Jena Bioscience, Jena Germany). For SYBR-green based quantitative real-time PCR (qRT-PCR) QuantiTect Primer assays (Qiagen) were analyzed in a CFX96 real-time detection system (Biorad). Probe-based assays (TaqMan Gene Expression Assays, ThermoFisher Scientific) were performed on a 7900HT Fast Real-Time PCR System (Applied Biosystems, ThermoFisher Scientific). ΔCT values were calculated relative to *hprt* expression. For the generation of transcriptomic profiles, 0.5 μg RNA of total lung lysates of 4 control or Tie2^cre^Rora^flox^ mice were pooled, respectively. RT2 Profiler PCR Array PAMM-074 (Qiagen) was performed according to manufacturer's instructions. To calculate relative expression of genes and to generate scatter-plots the web-based Data analysis center (Qiagen) was used.

### Histological Methods

Lung samples were fixed in Roti®-Histofix 4.5% (Carl-Roth, Germany) and embedded in paraffin. Six serial 3 μm slices of each lung sample were transferred on a microscope slide and subsequently stained either with hematoxylin and eosin (H&E) to examine immune cell infiltrates, tissue alterations and cryptococcoma formation or periodic acid Schiff's reagent (PAS) to visualize cryptococcal distribution and mucus production. Microscopy samples were analyzed on a Leica DMI 6000B.

### Magnetic Resonance Imaging (MRI) of Lungs *ex vivo*

MRI was performed on a 7 Tesla small animal MRI scanner (ClinScan 70/30; Bruker) using a whole body mouse volume coil (Bruker). For preparation of lung tissue for MRI analysis, total lungs were removed and fixed for 24 h in 4% paraformaldehyde (PFA; Merck,). Subsequently, fixed tissue was embedded in 50 ml Tubes (Sarstedt) containing 1.5% low-melting agarose (Sea Plaque agarose, FMC BioProducts) to avoid motion artifacts during MRI. The measurement protocol consisted of a T2 weighted Turbo-Spin-Echo (TSE) sequence with the following parameters: Time to repetition (TR): 4,000 ms, time to echo (TE): 74 ms, bandwidth (bw): 125 Hz/px, 20 averages, matrix: 512. For image post processing and data analysis, a dedicated software package (Chimera GmbH,) was used to segment hypointense lung nodules on the TSE sequence to determine the respective volumes in mm^3^. MRI was performed with the help of the Preclinical Imaging Platform Erlangen (PIPE).

### Enzyme-Linked Immunosorbent Assays (ELISA)

In order to determine lung tissue specific concentration of IL-4, IL-5, IL-13, IL-17, and IFN-γ, snap-frozen tissue pieces were homogenized in M-PER™ Mammalian Protein Extraction Reagent (ThermoFisherScientific), supplemented with PhosSTOP™ (Roche) and cOmplete™ protease Inhibitor cocktails (Roche). Protein concentration was determined with a Roti®-Quant protein quantification assay (Carl-Roth). For assessment of cytokine concentrations, 100 mg/ml total protein was used either IL-4 (eBioscience), IL-5 (Biolegend), IL-13 (eBioscience), IL-17 (Biolegend), or IFN-γ (eBioscience) specific ELISA assays. ELISA assays were carried out according to manufacturer's instructions.

### Statistics

Statistical tests were performed using Prism V7 (Graph Pad) software. If not otherwise indicated, a two-tailed Mann-Whitney *U* test with 95% confidence interval was performed for comparison of two groups (^*^P > 0.05; ^**^P > 0.01; ^***^P > 0.001; NS = not significant).

## Data Availability Statement

All datasets generated for this study are included in the article/Supplementary Material.

## Ethics Statement

The animal study was reviewed and approved by Regierung von Unterfranken.

## Author Contributions

MK, LK, and SO performed experiments and analyzed data. UM, GA, CB, US, and MN discussed the data and critically reviewed the manuscript. MK and SW conceptualized the project and wrote the paper.

### Conflict of Interest

The authors declare that the research was conducted in the absence of any commercial or financial relationships that could be construed as a potential conflict of interest.

## Supplementary Material

Supplemental Figure 1 **(A,B)** *In vitro* differentiated T cells obtained from control or Tie2^cre^Rora^flox^ mice. **(A)** Expression of T cell subpopulation defining transcription factors in differentiated T cells. **(B)** Relative abundance of *in vitro* differentiated T cell subpopulations. **(C,D)** Bone marrow chimeric C57BL/6 or Tie2^cre^Rora^flox^ mice were infected with 2,000 cfu of *C. neoformans* and analyzed 14 dpi. **(C)** Representative FACS plots and quantification of Lin^−^/Thy1^+^/ICOS^+^/KLRG1^+^ ILC2 in bone marrow chimeric mice (*n* = 4/group). **(D)** Fungal burden in lungs of infected bone marrow chimeric control and Tie2^cre^Rora^flox^ mice (*n* = 4/group). Data is expressed as mean ± SEM of **(A,B)** 3 or **(C,D)** one single experiment.

Supplemental Figure 2 **(A–D)** Control and Tie2^cre^Rora^flox^ mice were challenged with 500 cfu of *C. neoformans* and analyzed **(A,B)** 7 dpi or **(F,D)** 14 dpi. **(A)** Cryptococcal burden in infected lungs 7 dpi of control (*n* = 18) and Tie2^cre^RORa^flox^ (*n* = 20) mice assessed by plating serial dilutions of tissue homogenate on SAB-agar plates. **(B)** Representative H&E and PAS stainings of control and Tie2^cre^RORa^flox^ mice 7 dpi. **(C,D)** Fungal burden of control (*n* = 8) and Tie2^cre^RORa^flox^ (*n* = 9) mice in **(C)** brain and **(D)** liver 14 dpi determined by plating serial dilutions of tissue homogenates. Data is expressed as mean ± SEM pooled from **(A,C,D)** or representative **(B)** of two experiment.

Supplemental Figure 3 **(A–I)** Control and Tie2^cre^Rora^flox^ mice were challenged intranasally with 500 cfu *C. neoformans* and sacrificed **(A–F)** 7 dpi or **(G–H)** 14 dpi. **(A–F)** Quantification of pulmonary **(A)** CD11b^+^/CD64^−^/Ly6g^−^/SiglecF^+^ eosinophil granulocytes (eos), **(B)** CD11b^+^/Ly6g^+^/Ly6c^+^ neutrophil granulocytes (neu), **(C)** SiglecF^−^/MHCII^+^ cells, **(D)** SiglecF^−^/CD64^+^ cells, **(E)** SiglecF^−^/Ly6c^+^ cells and **(F)** CD11b^int/+^/CD11c^int/+^/CD64^+^/SiglecF^+^ alveolar macrophages in control (*n* = 10) and Tie2^cre^RORa^flox^ (*n* = 12) mice 7 dpi. **(G–I)** Quantification of **(G)** CD11b^+^/CD11c^+^/CD103^+^, **(H)** CD11b^+^/CD11c^+^/CD24^+^/Ly6c^−^ cDCs and **(I)** CD11c^+^/CD24^+^/Ly6c^+^ pDCs in lungs of infected control (*n* = 8) and Tie2^cre^RORa^flox^ (*n* = 9) mice 14 dpi. Data is expressed as mean ± SEM pooled from two experiments.

Supplemental Figure 4 Gating strategies used in flow cytometry. **(A)** Gating strategy for Lin^−^/Thy1^+^/ICOS^+^/KLRG1^+^ ILC2 in *C. neoformans* infected control and Tie2^cre^Rora^flox^ mice 14 dpi. **(B)** Gating strategy for CD11b^+^/CD64^−^/Ly6g^−^/SiglecF^+^ eosinophilic granulocytes (eos), CD11b^+^/Ly6g^+^/Ly6c^+^ neutrophil granulocytes (neu), SiglecF^−^/MHCII^+^ cells, SiglecF^−^/CD64^+^, SiglecF^−^/Ly6c^+^, and CD11b^int/+^/CD11c^int/+^/CD64^+^/SiglecF^+^ alveolar macrophages (aMϕ) representative shown for control mice 14 dpi after *C. neoformans* infection.
