# Supplementary figures and images for: Group 2 Innate Lymphoid Cells (ILC2) Suppress Beneficial Type 1 Immune Responses During Pulmonary Cryptococcosis
Source: Front Immunol. 2020 Feb 14;11:209. doi: 10.3389/fimmu.2020.00209 (PMC7034304; doi:10.3389/fimmu.2020.00209)

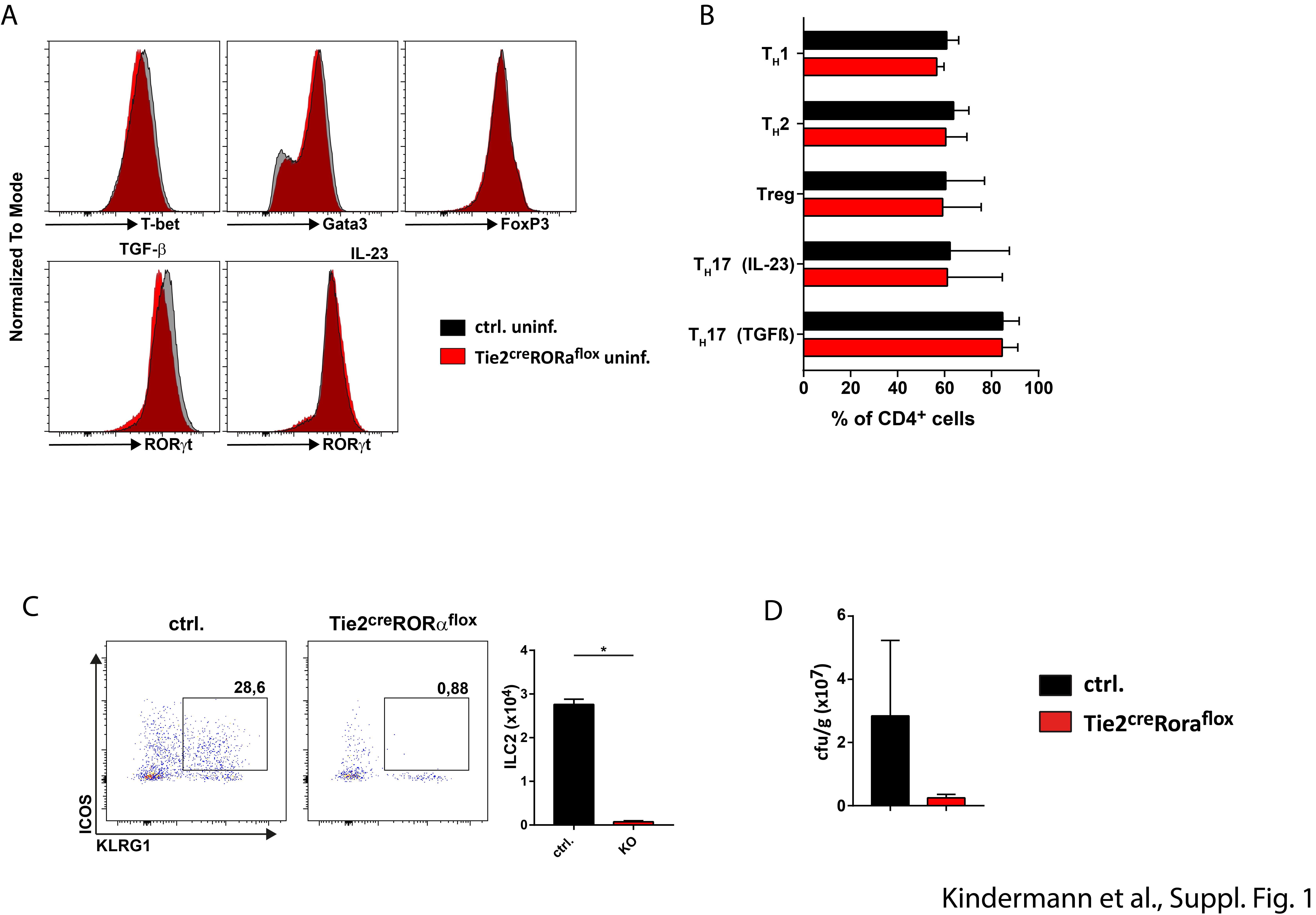

Supplement: Supplemental Figure 1 — (A,B) In vitro differentiated T cells obtained from control or Tie2creRoraflox mice. (A) Expression of T cell subpopulation defining transcription factors in differentiated T cells. (B) Relative abundance of in vitro differentiated T cell subpopulations. (C,D) Bone marrow chimeric C57BL/6 or Tie2creRoraflox mice were infected with 2,000 cfu of C. neoformans and analyzed 14 dpi. (C) Representative FACS plots and quantification of Lin−/Thy1+/ICOS+/KLRG1+ ILC2 in bone marrow chimeric mice (n = 4/group). (D) Fungal burden in lungs of infected bone marrow chimeric control and Tie2creRoraflox mice (n = 4/group). Data is expressed as mean ± SEM of (A,B) 3 or (C,D) one single experiment. [file Image_1.jpeg]

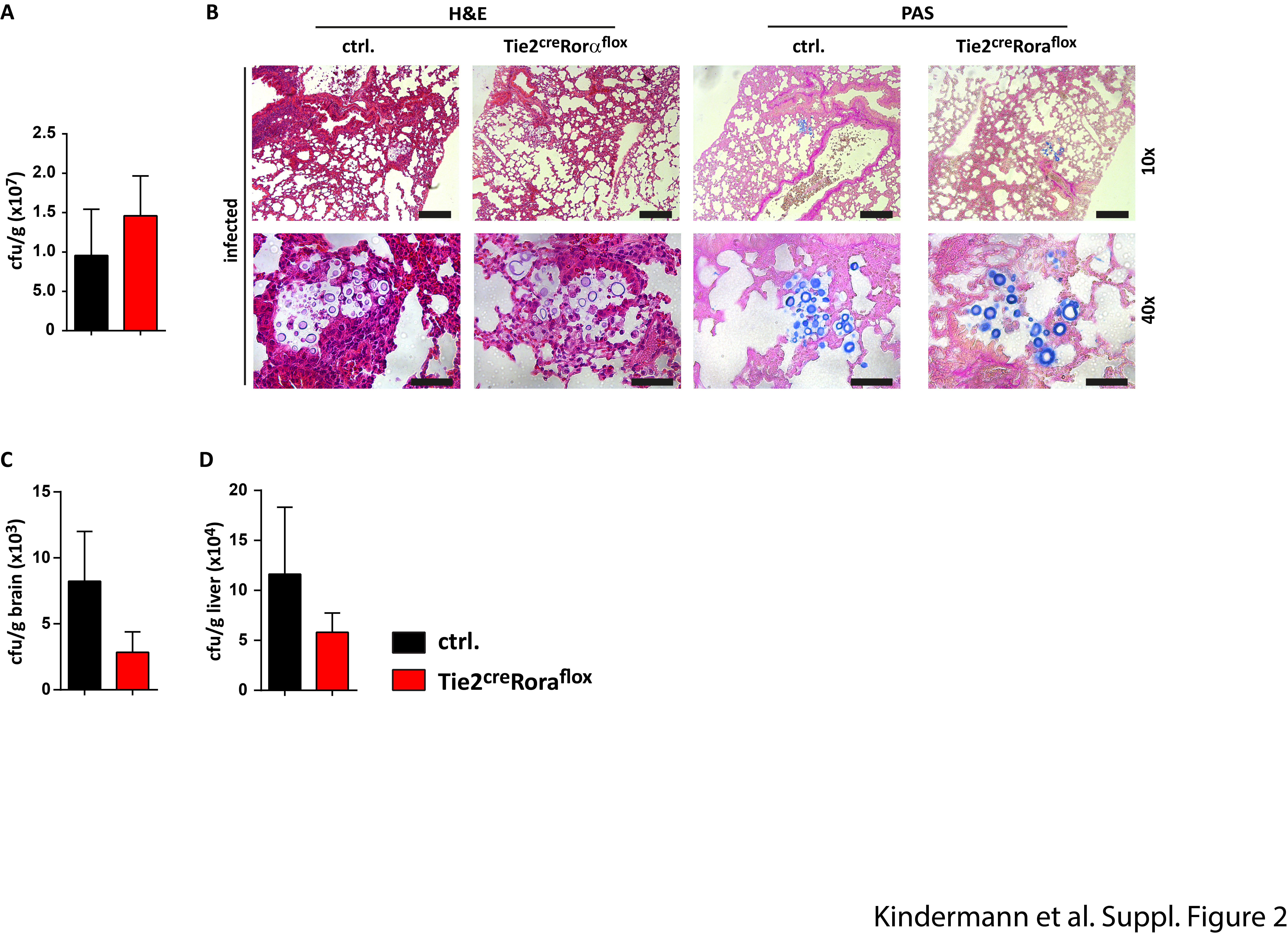

Supplement: Supplemental Figure 2 — (A–D) Control and Tie2creRoraflox mice were challenged with 500 cfu of C. neoformans and analyzed (A,B) 7 dpi or (F,D) 14 dpi. (A) Cryptococcal burden in infected lungs 7 dpi of control (n = 18) and Tie2creRORaflox (n = 20) mice assessed by plating serial dilutions of tissue homogenate on SAB-agar plates. (B) Representative H&E and PAS stainings of control and Tie2creRORaflox mice 7 dpi. (C,D) Fungal burden of control (n = 8) and Tie2creRORaflox (n = 9) mice in (C) brain and (D) liver 14 dpi determined by plating serial dilutions of tissue homogenates. Data is expressed as mean ± SEM pooled from (A,C,D) or representative (B) of two experiment. [file Image_2.jpeg]

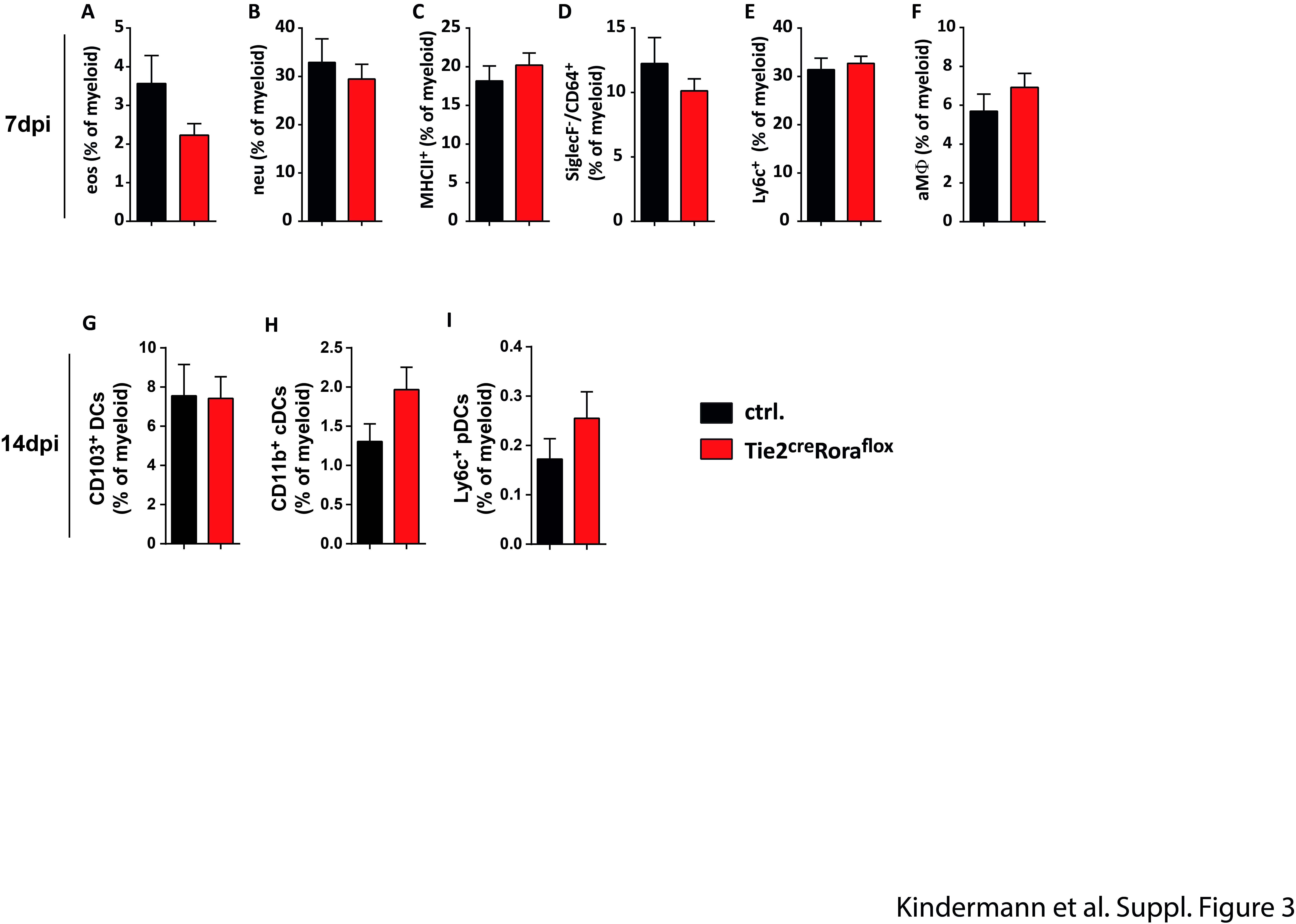

Supplement: Supplemental Figure 3 — (A–I) Control and Tie2creRoraflox mice were challenged intranasally with 500 cfu C. neoformans and sacrificed (A–F) 7 dpi or (G–H) 14 dpi. (A–F) Quantification of pulmonary (A) CD11b+/CD64−/Ly6g−/SiglecF+ eosinophil granulocytes (eos), (B) CD11b+/Ly6g+/Ly6c+ neutrophil granulocytes (neu), (C) SiglecF−/MHCII+ cells, (D) SiglecF−/CD64+ cells, (E) SiglecF−/Ly6c+ cells and (F) CD11bint/+/CD11cint/+/CD64+/SiglecF+ alveolar macrophages in control (n = 10) and Tie2creRORaflox (n = 12) mice 7 dpi. (G–I) Quantification of (G) CD11b+/CD11c+/CD103+, (H) CD11b+/CD11c+/CD24+/Ly6c− cDCs and (I) CD11c+/CD24+/Ly6c+ pDCs in lungs of infected control (n = 8) and Tie2creRORaflox (n = 9) mice 14 dpi. Data is expressed as mean ± SEM pooled from two experiments. [file Image_3.jpeg]

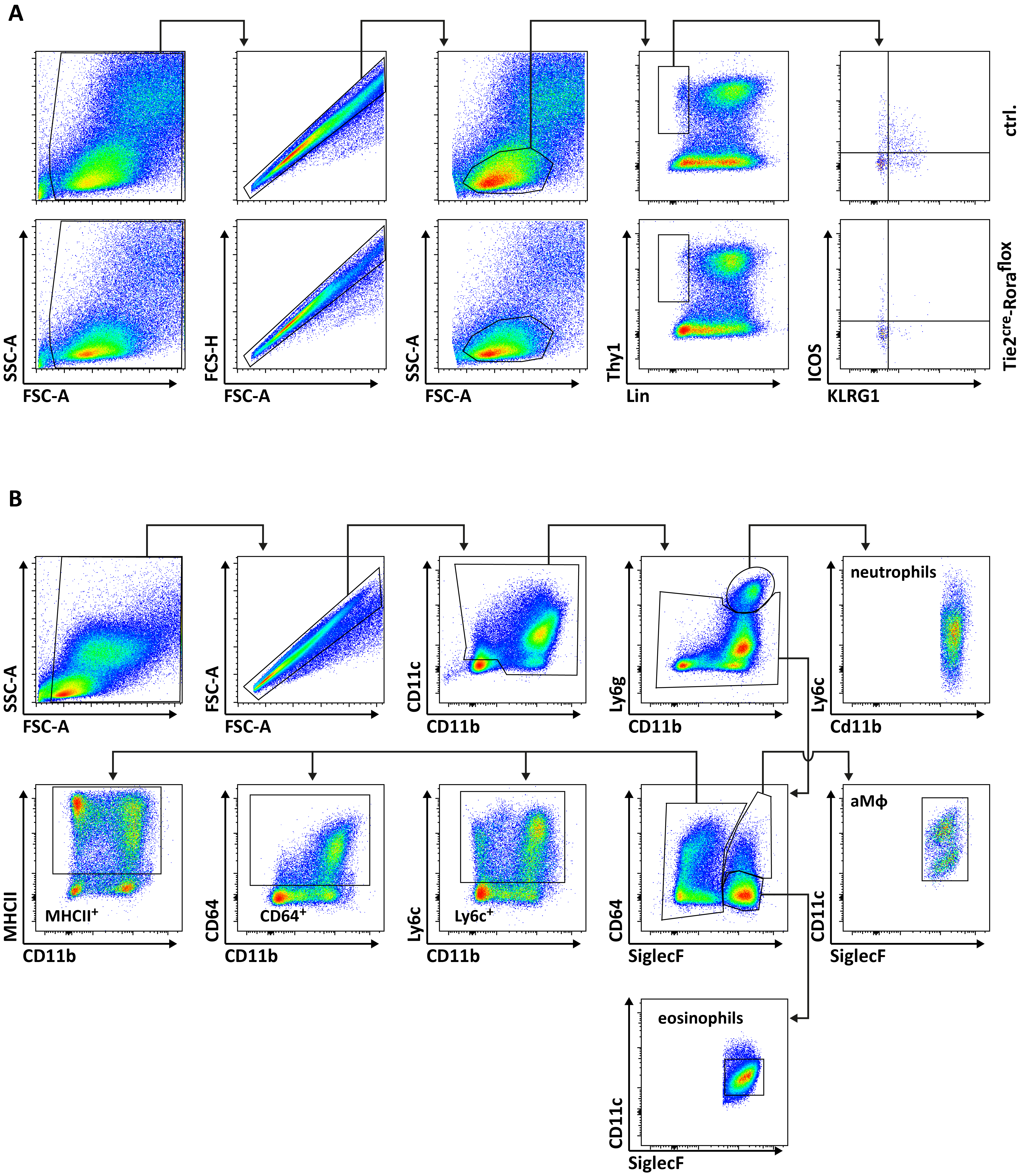

Supplement: Supplemental Figure 4 — Gating strategies used in flow cytometry. (A) Gating strategy for Lin−/Thy1+/ICOS+/KLRG1+ ILC2 in C. neoformans infected control and Tie2creRoraflox mice 14 dpi. (B) Gating strategy for CD11b+/CD64−/Ly6g−/SiglecF+ eosinophilic granulocytes (eos), CD11b+/Ly6g+/Ly6c+ neutrophil granulocytes (neu), SiglecF−/MHCII+ cells, SiglecF−/CD64+, SiglecF−/Ly6c+, and CD11bint/+/CD11cint/+/CD64+/SiglecF+ alveolar macrophages (aMϕ) representative shown for control mice 14 dpi after C. neoformans infection. [file Image_4.png]
